# Establishing a new journal for systematic review products

**DOI:** 10.1186/2046-4053-1-1

**Published:** 2012-02-09

**Authors:** David Moher, Lesley Stewart, Paul Shekelle

**Affiliations:** 1 Clincal Epidemiology Program, Ottawa Hospital Research Institute, The Ottawa Hospital - General Campus, 501 Smyth Road, Box 201B, Ottawa, ON K1H 8L6, Canada; 2 Department of Epidemiology & Community Medicine, Faculty of Medicine, University of Ottawa; 3 Centre for Reviews and Dissemination (CRD), University of York, UK; 4 West Los Angeles VA Medical Center, Los Angeles, CA 90066 USA

**Keywords:** new journal, systematic reviews, open access

## Abstract

Welcome to a new age in publishing systematic reviews. We hope the launch of *Systematic Reviews *will resonate with a broad spectrum of readers interested in using them in a variety of ways, such as providing comprehensive and up to date evidence for patient management, informing health policy, and developing rigorous practice guidelines. Systematic reviews are increasingly popular. Our journal is committed to publishing a wide variety of well conducted and transparently reported systematic reviews and associated research. We are open access and electronic and not confined by space and so offer scope for publishing reviews in detail and providing a modern and innovative approach to publishing. We look forward to participating in the voyage with all of our readers.

## 

Welcome to *Systematic Reviews*. You may be already familiar with systematic reviews, or be curious and have accessed our journal to learn more about them.

A systematic review is a review "of a clearly formulated question that uses systematic and explicit methods to identify, select, and critically appraise relevant research, and to collect and analyze data from the studies that are included in the review. Statistical methods (meta-analysis) may or may not be used to analyze and summarize the results of the included studies." [1].

There are several reasons underlying the need for systematic reviews. Given the vast amount of information published in the biomedical literature, it is almost impossible to keep up to date by reading reports of individual studies - the trajectory of research publications is variable and very frequent in specific healthcare areas. But, individual studies are seldom sufficient to drive change. They are often too small to reach reliable conclusions, and for fair evaluation, it is important to look at the totality (or at least an unbiased sample of the totality) of evidence in favour of, against, or neutral to the healthcare intervention under consideration. Systematic reviews provide a means of doing this in an objective, transparent and reproducible way. With a well developed question, sound methods, the results of systematic reviews provide strong evidence for rational decision making.

Systematic reviews emerged in healthcare in the 1980s after initial development in the fields of psychology and education [2]. The 1990s saw many important developments, including the establishment of the Cochrane Collaboration, a network of about 28,000 professionals dedicated to synthesizing the effectiveness of interventions across all of healthcare. Systematic reviews became firmly embedded in UK health decision making [3] and the Evidence-based Practice Centre (EPC) program, a network of 14 centres throughout North America, was also established during the 1990s [4].

Today the science and use of systematic reviews is firmly established. In many countries systematic reviews are used extensively in setting health policy, either directly to help inform healthcare decision making at organisational and governmental levels, or via their use in the development of practice guidelines. Clinicians read systematic reviews as an efficient way to help keep up with the literature for patient management [5] and that patient summaries prepared from EPC program are viewed hundreds to thousands of times a month [6]. The U.S. Institutes of Medicine has judged that practice guidelines can only be considered trustworthy if they are based on a systematic review of the evidence.

More than 5000 systematic reviews are indexed annually in Medline [7]; one recent estimate is that 11 new systematic reviews in healthcare are published daily [8]. Despite this volume there is no open access journal devoted broadly to publishing high quality systematic review products. *Systematic Reviews* will fill this gap and will consider for publication well conducted and transparently reported reviews, irrespective of their findings. These reviews maybe traditional systematic reviews, review of systematic reviews (overviews), individual patient data meta-analysis, and other types of systematic reviews. We are also committed to publishing papers that address methods underpinning how systematic reviews are conducted and reported.

We will also publish protocols of systematic reviews, providing a full public record of the rationale for a review and the methods that are planned. We anticipate a growing number of published protocols as the recently established PROSPERO register of systematic review protocols gathers momentum (http://www.crd.york.ac.uk/prospero) and [7,8]. Protocols submitted for publication consideration that are registered and externally funded will receive an expedited peer review. We are committed to working towards the universal registration of systematic reviews. Several articles in this inaugural issue of *Systematic Reviews *are devoted to talks presented at the PROSPERO launch meeting in 2011 http://www.kscanada.ca/.

There is a general consensus regarding how reviews can best address certain questions, such as what are the comparative long-term benefits and harms of co-administration of different lipid-modifying agents for patients who require intensive lipid-modifying therapy, (i.e., a statin plus another lipid-modifying agent) compared with higher dose statin monotherapy. However, newer methods of review and synthesis continue to evolve to help answer more complex questions. For example, providing there is a good deal of similarity or exchangeability across a body of research studies, network meta-analysis allows comparison of the effectiveness of interventions that have not been compared directly, such as the relative benefits of nonhormonal treatments for advanced breast cancer [10]. Methods are also being developed and refined to synthesise diagnostic and prognostic studies and to address other areas of health, such as understanding the policy implications of needle exchange clinics.

Keeping systematic reviews up-to-date is important to policy makers and practitioners, but sometimes there is little enthusiasm for submitting updates for publication because most journals are reluctant to publish them. There have been some attempts to resolve this issue [11]. Our approach will be to embrace the publication of updates (with appropriate disclosure and links to previous versions) and to ensure that any published systematic review update gets a unique citation. We hope this will make publishing attractive to authors and in consequence will provide the community with easy access to the most up to date results. We are also working to ensure a reduced article processing charge (APC) for updates of less than 2000 words and 15 references. A similar approach to publishing research and protocol updates was recently introduced by our sister publication, *Trials *(http://www.trialsjournal.com), with an update on the Third International Stroke Trial (IST-3) [12].

We have a strong commitment to ensuring that all reviews we publish are reported completely and accurately. Use of reporting guidelines appears to be associated with increased quality of reporting [13,14] and we therefore recommend that authors and peer reviewers use appropriate reporting guidelines. We similarly ask peer reviewers to use reporting guidelines to help guide their peer review, and as editors we will use reporting guidelines to help make decisions about the acceptability of manuscripts to be published in the journal. In the first instance the PRISMA statement [15,16], a reporting guideline for systematic reviews and meta-analyses of healthcare interventions should be followed for reporting reviews submitted to the journal. We will alert readers about additional reporting guidance when they become available.

For some types of systematic reviews, such as rapid reviews and updates of original reviews or previously updated reviews, there is no standard as to how best they should be presented. We encourage authors to innovate.

*Systematic Reviews* is an electronic journal. No print issues will be available. Beyond being eco-friendly, electronic publishing offers several advantages including speed to publication and unlimited space for complete reporting (references, tables, figures, and additional files). This will help us realize another important goal of the journal, namely, getting the evidence to those who need to know about it as quickly as possible. Electronic threading or linking, particularly for systematic review protocols (to publication of complete review) and updates (to complete reviews and/or updates of previous updates) should also be a useful feature for readers in the future - innovations which our publisher, and *Trials*, are currently developing [17].

Being open access is important to us. It enables free access of all journal content to interested readers, globally. Authors retain copyright on their research, and our publisher is committed to archiving, appropriately, all published pages for future use.

We are working with our publisher to help ensure *Systematic Reviews *stays as cutting edge as possible in how it delivers knowledge to readers. One idea is that for protocols registered in PROSPERO or published in the journal readers can sign up to be notified when the completed review is published. We will also offer this service for updates of already completed reviews published in the journal. This service will happen initially for systematic reviews published across the BioMed Central platform. We intend to establish a 'systematic review of the year' competition and one for the best example of how a review impacted practice.

We have also worked with our publisher to develop incentives to publish in *Systematic Reviews*. Authors who publish protocols in the journal will be offered a 20% reduced article processing charge (APC) to publish the completed review. Similarly, authors of completed reviews will be offered a similar reduced APC when publishing updates of completed reviews in the journal.

We will also use our journal to alert readers about interesting systematic review products and planned and ongoing activities in the systematic review community. We will provide a regular electronic update of new systematic reviews registered in PROSPERO and of new systematic reviews included in DARE (Database of Abstracts of Reviews of Effects) [18]. We will also publish a calendar of conferences and events related to systematic review and invite notification of such events.

We encourage readers to let us know about other innovations they think we should consider. *Systematic Reviews* will succeed if there is an active and innovative partnership between us and our readers and we hope that the journal will become a focus for communication and forum for debate across the systematic review community. We look forward to publishing a broad range of systematic review products and collaborating with all of you in the coming years.
